# Approach To The First Unprovoked Seizure- PART I

**Published:** 2013

**Authors:** Mohammad GHOFRANI

**Affiliations:** 1 Pediatric Neurology Research Center, Shahid Beheshti University of Medical Sciences, Tehran, Iran; 2 Pediatric Neurology Department, Mofid Children Hospital, Faculty of Medicin, Shahid Beheshti University of Medical Sciences, Tehran, Iran

**Keywords:** First, Unprovoked, Seizure, Children, Anti Epileptic Drugs (AED), Treatment

## Abstract

The approach to a child who has experienced a first unprovoked generalized tonic-clonic seizure is challenging and at the same time controversial.

How to establish the diagnosis, ways and means of investigation and whether treatment is appropriate, are different aspects of this subject.

In this writing the above mentioned matters are discussed.

## Introduction

The approach to a child who has experienced a first unprovoked generalized tonoclonic seizure is an important endeavour in daily clinical pediatric neurology practice.

If occurrence of an ictal event is established, the main question is whether treatment by Anti Epileptic Drugs (AED) should be initiated or not?

The main reason for prescribing AED is to prevent further seizure. Thus such therapy is justified when there is reasonable chance seizure will recur. Deciding to initiate treatment requires balancing the risk of drug side effects against the psychosocial consequences of future convulsions.

Knowing that treatment does not ensure that seizure will not recur and that it merely lowers the probability of recurrence, it behooves us to think twice about initiating AED therapy.

As we discuss the subject of first unprovoked seizure (FUS) it is necessary to have a clear-cut understanding of FUS.

A seizure is defined as abnormal paroxysmal neuronal discharge which is clinically manifested by motor, sensory, autonomic or behavioral disturbances (1).

We hypothesize that ictal event is secondary to an imbalance between excitatory and inhibitory neurotransmitter activities in the brain. A provoked seizure is characterized by a specific trigger such as fever, central nervous system infection, intoxication or head injury. In these situations, it is definitely indicated to treat the seizure immediately along with addressing the triggering cause(s).

On the contrary, unprovoked seizure is not associated with an obvious precipitating cause and may be related to epilepsy. Some authorities believe the overall recurrence risk for another seizure after a first unprovoked episode is 45% (22% at 6 months, 29% at 12 months, 37% at 24 months, 43% at 60 months and 46% at 120 months) (1).

Population-based studies of the incidence of first unprovoked seizure suggest that there are between 25,000 and 40,000 children per year in the United State who experience a first unprovoked seizure (2-6).

Until recently, it was common practice for practitioners to prescribe a long duration of daily antiepileptic drug (AED) therapy after a child or adolescent experienced a single seizure of any type. The rationale for this practice was the belief that all seizures were likely to recur and that seizure could be detrimental, causing brain insult. Also, it was thought that if any recurrence were to take place, this would lead to progressively more seizures.

It was also assumed that AEDs were safe, having few side effects and were effective in prevention of seizure recurrence (2). These assumptions have undergone significant change over the past two decades, leading to a more optimistic view about the nature of seizure and a more conservative approach to the use of treatment (2). Recognition of different settings in which an unprovoked seizure occurs and identification of risk factors for recurrence helps us define the appropriate management. After a first unprovoked seizure, the decision regarding starting antiepileptic drug (AED) therapy, should be made on the basis of balancing the risk of side effects of AED versus seizure recurrence(1). Neonatal seizures are not considered part of this subject. Many children are seen by a physician after a first unprovoked generalize tonic-clonic seizure, a few after a first complex partial seizure, but almost none after a single absence or myoclonic seizure (7). When a child presents after a single unprovoked seizure, the question which is immediately raised is will it happen again?

Beregr and Shennar in their meta-analysis of the recurrence risk after a first seizure concluded that overall about 40 percent will have another seizure (7).

Seizure prevention has been a concern even since Gowers wrote” the tendency of the disease is to self perpetuation; each attack facilitates the occurrence of another, by increasing the instability of nerve element” (8). Animal studies on kindling, which is an experimental technique for creating epilepsy by a series of subclinical electrical stimulations of the temporal lobe, induces progressive intensification of electrographic and behavioral seizures (9-11).

Also we have evidence from animal studies that prolonged or repeated convulsions under special situations can induce neuronal damage and predispose to epilepsy (12).

In affirmation of these evidences, recent animal research showed that prolonged convulsions, occurring during critical periods of brain development may alter neuronal activity and circuitry which predispose to future epilepsy (2, 13-15). We do not know how relevant animal studies are to seizure in human beings and at this point we are reluctant and skeptical in expanding the results of animal models to our daily clinical practice (2, 15). At the same time clinical evidence in pediatric neurology indicates that even prolonged seizure seldom causes clinically discernible brain injury unless associated with an acute neurologic insult (16).

## Why to treat

The main reason for initiating treatment is concern for the risk of physical injury or death from a subsequent seizure, Serious injury from a seizure in a child is a rare event, which may occur after a fall associated with loss of consciousness. To reduce that risk, restrictions are recommended that would apply to any young child, such as bicycling on a sidewalk rather than the street and always with a helmet and swimming only with a buddy (2).

Showering rather than bathing is recommended for children and adolescents, unless they are supervised. Sudden unexpected death in children with epilepsy is, fortunately, very uncommon. When death occurs in epileptic children, it is nearly always related to an underlying neurologic or cardiac problem rather than epilepsy (2, 12-19).

One-population-based study found that the risk of death in those with childhood-onset epilepsy is the same as that for the general population of children without significant neurologic disorder (2, 20). So far, no studies have been found that examined whether treating a child after a first unprovoked seizure would decrease the likelihood of either subsequent significant injury or sudden death.

## Psychological Considerations

It should be known that taking daily AED is not an easy act.

The effect of taking daily medication on the child’s self esteem should be a concern (21). A child who is taking medication for long duration is perceived to have a long standing illness by the child, family and school. Additionally, chronic treatment for seizure prevention is a burden for the family and may affect the ability to obtain health insurance. Issues in teenagers become more complicated as concerns about driving license and teratogenicity come into play (2).

## Was the event really a seizure?

The essential question that is raised when we face a child who allegedly experienced a first unprovoked generalized tonoclonic convulsion is: was the event a seizure? On many occasions the first challenge is differentiating between a true seizure and seizure mimickers. Syncope, breath holding spells, tics and other movement disorders and night terrors are a few examples. Careful description of events by a reliable person is of great value. Precipitating events, warning symptoms, duration, semiology of seizure and description of the postictal period are crucial aids in the characterization of an event (1).

Taking into consideration the possibility that the event was not the first one is as significant as identifying a true seizure from others paroxysmal phenomena. With careful questioning, retrospective recognition of a previous nonconvulsive or convulsive event is possible. Having this information may change the approach because a child who has had at least two unprovoked seizures is perceived as epileptic. If this is the case, and depending on successful classification of the epilepsy syndrome, the decision to treat or not to treat may be less controversial. The risk of further seizures may outweigh the adverse effects of antiepileptic drugs (1). When diagnosis of a seizure is reached, the next step is to determine the etiology. Febrile state, trauma, intercurrent infection, if present, preclude the diagnosis of an unprovoked seizure. The neurologic examination may provide important clues in regard to the etiology of the seizure. Finding hypopigmented spots, focal neurologic deficit, evidence of mental retardation or cerebral palsy, suggest a symptomatic cause and dismiss unprovoked seizure.

## What tests should be obtained

The kind of tests which have to be performed should be individualized, oriented toward each particular case. Complete Blood Cell count and basic chemistry panel should be done only under specific clinical circumstances. Whenever an infectious process is suspected, C.B.C. examination is justified. A Glucose level should be obtained in a diabetic patient and BUN/Creatinine and basic electrolytes are of significance in the patients with history of renal disease, vomiting or diarrhea. A lumber puncture is indicated whenever meningeal signs are present or in very young children, in whom meningeal signs may not be significant.

Lumbar puncture is also recommended if a child who had a first unprovoked seizure remains with diminution of level of consciousness for a long period which cannot be attributed to postictal state. In the majority of circumstances, the caretaker physician may prefer to perform a cranial neuroimaging examination, especially if the child has to have a lumbar puncture examination. In these cases, by performing cranial neuroimaging, the probability of increased intracranial pressure is investigated.

## What type of neuroimaging is preferred?

Early reports in the era shortly after the introduction of computed tomography (CT) showed that close to 30% of children with refractory epilepsy had imaging abnormalities (22-23).

Many of these children were known to be neurologically abnormal. With the availability of high quality magnetic resonance imaging (MRI) and increased sensivity of MRI, nowadays we are able to have an in vivo view of pathological anatomy as well as to detect lesions such as migration defects and mesial temporal sclerosis, both of which are known to cause childhood onset seizure. It is clear that these two abnormalities are not readily seen on CT. Also we are aware of otherwise normal patients with normal CT scans whose brain lesions were detected by MRI (24-26).

In the U.S. and Europe, adults who present with seizure are usually candidates for a neuroimaging study. (22, 27-29). However, in the pediatric age group, the decision is less clear due to the overall lower rate of tumor, the previously low yield on CT, and the need to sedate younger children in order to perform the MRI (24).

The International League Against Epilepsy (ILAE, 1997) has recommended neuroimaging with an MRI when feasible for all epileptic patients, including children, who do not have a clear cut identifiable idiopathic epilepsy syndrome (22). However, the decision is less clear after a first unprovoked seizure which may turn out to be an isolated event without any recurrence. A published Practice Parameter of the American Academy of Neurology, American Epilepsy Society and Child Neurology Society on the evaluation of a first nonfebrile seizure in children lists neuroimaging as a practice option due to the paucity of available information to support its use (30). In a prospective study, 411 children with a first unprovoked afebrile seizure seen at Montefiore Medical Center, Bronx, New York, between October 1983 and August 1992 were enrolled and followed. Neuroimaging studies were performed in 218 (53%) of the 411 children in the first seizure study. Abnormalities were found in 45 (21%). Of the 218 children who had neuroimaging studies, 159 (73%) had a CT only, 32 (15%) had an MRI only and 27 (12%) had both a CT and an MRI performed. Of the 27 children who had both CT and MRI, the MRI showed an abnormality either not seen on CT or different than that seen on the CT in 8 cases. In the 32 children who only had an MRI, 16% were abnormal. The types of abnormalities found can be divided into abnormalities that altered both the diagnosis and the acute management, abnormalities that provided additional data regarding etiology and/or localization in a child with a first unprovoked seizure, and abnormalities that were probably incidental (22). Four children who clinically were felt to have a first unprovoked seizure were found to have lesions which altered acute management and which excluded them from the first seizure study. These included two children with neuroimaging evidence of neurocysticercosis and two with tumors; all of these children had indications for neuroimaging.

The two children ages 8 and 11 with neurocysticercosis both presented with convulsive status epilepticus lasting over 30 minutes. In addition, seizure was clearly focal in the 8 year old.

The 4 year old with a medullablostoma presented with staring and a respiratory arrest and had an abnormal neurological examination after the seizure, which prompted the emergency CT scan. The 17 year old with ganglioglioma presented in 1985 with a focal seizure,which then evolved to generalized seizure lasting 3 minutes. All of these four cases, had clinical reason for neuroimaging, including a respiratory arrest and abnormal neurological examination, status epilepticus in two cases, and a focal seizure in three cases. In summary, of the 411 children with an apparent FUS, 218 had an imaging study of which 45 (21%) were abnormal. The most common abnormalities included: focal encephalomalacia, cerebral dysgenesis and other focal findings. Nine children were found to have mesial temporal changes on MRI.
